# Onset of HLA-B27-associated diseases in diabetic patient during a period of religious fasting: A case report

**DOI:** 10.1097/MD.0000000000010104

**Published:** 2018-03-16

**Authors:** Zhenyun Lin, Binbin Zhu, Xiuming Jin

**Affiliations:** a Department of Obstetrics and Gynecology, Hangzhou Maternity Hospital; b Eye Center, Second Affiliated Hospital of Zhejiang University School of Medicine, Hangzhou, China.

**Keywords:** acute anterior uveitis, fasting, HLA-B27, ulcerative colitis and ankylosing spondylitis

## Abstract

**Rationale:**

The association between human leukocyte antigen B27 (HLA-B27) with its associated diseases is far from complete. The role of HLA-B27 in disease susceptibility is still not known, although many suggestions have been proposed.

**Patient concerns:**

The patient was a 46-year-old policeman with a history of obesity, diabetes, and hypertension. He was a Shaolin lay disciple who fasted at the Shaolin temple for at least 1 week each year since 2014.

**Diagnoses:**

The patient suffered three different HLA-B27-associated diseases including acute anterior uveitis, ulcerative colitis, and ankylosing spondylitis, from 2014 to 2016 because of prolonged fasting.

**Interventions:**

The patient accept standard treatment after the diagnosis of acute anterior uveitis, ulcerative colitis, and ankylosing spondylitis.

**Outcomes:**

The patient's symptoms and signs of acute anterior uveitis, ulcerative colitis, and ankylosing spondylitis were all relieved within one week after the clinical treatment.

**Lessons:**

Our case suggested that prolonged fasting may lead to the onset of HLA-B27-associated diseases in diabetic patient.

## 1 Introduction

The association between human leukocyte antigen B27 (HLA-B27) and its associated diseases is unclear. Moreover, the role of HLA-B27 in disease susceptibility is unclear even though several studies have provided many suggestions. In this report, we present the case of a diabetic patient who developed three HLA-B27-associated diseases from 2014 to 2016 because of prolonged fasting for 1 week each year.

## 2 Case presentation

This case report conformed to the principles of the Declaration of Helsinki. Study procedures were approved by the Ethics Committee of the Affiliated Second Hospital, School of Medicine, Zhejiang University, China. Written informed consent was obtained from the patient for data collection and subsequent analyses. The patient was a 46-year-old policeman with a history of obesity, diabetes, and hypertension. He was a Shaolin lay disciple who fasted at the Shaolin temple for at least 1 week every year since 2014. During the fasting period, he did not consume anything except water. On November 27, 2014, the patient was brought to the emergency department of our hospital after 5 days of fasting and was diagnosed with HLA-B27-positive ankylosing spondylitis (AS). AS is a chronic multisystem inflammatory disorder primarily involving the sacroiliac joints and axial skeleton. AS was diagnosed by a rheumatologist according the medical history, signs, and symptoms of the patient.^[1]^ The patient was administered mesalazine (4 g/day) and progressed favorably, with a good response after 5 days. On December 12, 2015, the patient was diagnosed with ulcerative colitis (UC) immediately after the fasting period. UC causes inflammation and ulcers in the lining of the large intestine and usually affects the lower section (sigmoid colon) and the rectum. UC was diagnosed by a digestive physician according to the medical history, signs, and symptoms of the patient.^[2]^ The patient was administered oral mesalazine (4 g/day) and his symptoms were relieved after 1 week. Interestingly, the patient was diagnosed with acute anterior uveitis (AAU) of the left eye on December 14, 2016, that is, 5 days after the fasting period. AAU was diagnosed by an oculist according the signs of AU, including dilation of ciliary vessels, the presence of cells and flare in the anterior chamber, and keratic precipitates on the posterior surface of the cornea.^[3]^ The patient was administered 0.1% TobraDex every 2 hours and 0.1% pranpulin 4 times a day in both the eyes. Moreover, he was administered 0.5% atropine 3 times a day. After 1 week, cellular reactions and conjunctival vessel engorgement subsided, synechiae of the left eye were resolved, and final visual acuity was restored to 20/20 in both the eyes. Assessment of the patient after 1 month did not show the recurrence of inflammation. After each treatment, the patient successfully lost weight; moreover, his blood pressure and glucose levels were well controlled without any medication after prolonged fasting (Table 1).

**Table 1:**
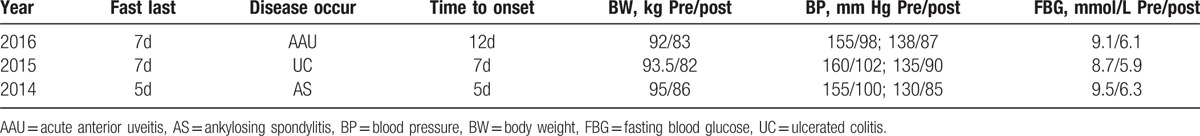
Data of the diabetic patient with HLA-B27 associated diseases as a result of prolonged fasting.

## 3 Discussion

HLA-B27 plays an important role in the pathogenesis of HLA-B27-associated diseases; however, different diseases may develop along different lines. Moreover, 3 different HLA-B27-associated diseases have been rarely reported in the same patient, further confirming that UC, AS, and AAU may be induced by different clinical manifestations of HLA-B27 abnormity. Previous studies suggest that immune-mediated diseases, similar to those associated with the overlapping of susceptibility genes, may involve overlapping pathogenic pathways.^[4]^ Halling et al^[5]^ reported that various immune-mediated diseases frequently develop in patients with inflammatory bowel disease (IBD).

Although several studies performed over 30 years have focused on the role of HLA-B27, none of these studies have been able to prove or discard the postulated mechanisms of action of HLA-B27.^[6]^ Approximately 40% patients with AS develop sudden-onset unilateral AU sometime during the course of their disease. Therefore, patients with AAU, especially HLA-B27-positive patients, should be examined for inflammatory low back pain and for other clinical features of spondyloarthropathy. Our experience with this case suggests that prolonged fasting induces HLA-B27-associated diseases in diabetic patients. Preliminary data of a previous study ^[7]^ suggest that HLA-B27 misfolding activates IL-23/IL-17 axis and that this novel mechanism may at least partly explain the role of HLA-B27 in the pathogenesis of colitis in transgenic rats. Various drugs, including antibiotics, statins, methotrexate, thiopurines, and biological agents, are suggested to induce autoimmunity.^[8–10]^ Prolonged fasting may induce fat, sugar, and protein metabolic disorders in diabetic patients. These metabolic disorders may induce HLA-B27 misfolding, subsequently leading to HLA-B27-associated diseases. In addition, metabolic inflexibility-associated “immunological fitness” induced by fasting may promote HLA-B27-associated diseases.^[11]^

Nonsteroidal anti-inflammatory drugs (NSAIDs) are the first-line treatment for AS and UC. Moreover, numerous studies have shown the ability of NSAIDs to provide rapid relief from inflammatory back pain.^[12]^ A Cochrane report indicates that high-dose mesalazine is effective for treating mild-to-moderate UC.^[13]^ Intensive topical steroids with mydriatics are recommended for treating AAU.

Chinese traditional medicine suggests that Bigu (refraining from eating grains) does not harm the human body.^[14]^ However, our findings suggest that prolonged fasting by diabetic patients may induce HLA-B27-associated diseases. Therefore, additional studies should be performed to determine the relationship between prolonged fasting and general disease pathogenesis in diabetic patients.

## 4 Author contributions

**Conceptualization:** Z. Lin.

**Investigation:** B. Zhu, X. Jin.

**Methodology:** B. Zhu, X. Jin.

**Resources:** X. Jin.

**Validation:** Z. Lin.

**Writing – original draft:** Z. Lin.

**Writing – review & editing:** X. Jin.
